# Quantitative levels of interferon gamma as a biomarker in the aqueous and serum samples of infectious and noninfectious uveitis patients

**DOI:** 10.1186/s12348-025-00461-1

**Published:** 2025-02-26

**Authors:** Sahar Saad Eldeen Mohamed Shaban, Hend Mohammed Safwat, Abeer Mohammed Abdelmohymen, Donia Ahmed Hassan, Mona Nabeh Mansour, Hanan Said Mohammed, Nora Seliem, Shahinaz El Attar, Doaa Refaat Amin, Seham Kamal Ahmed Khirala, Aya Ahmed Ghamry, Mona Gamal El Din Al Anany, Shaymaa Fathy Mohammed

**Affiliations:** Faculty of Medicine (for Girls), Al-Azhar University, Cairo, Egypt

**Keywords:** Uveitis, Biomarker, Infectious, Noninfectious, Interferon gamma

## Abstract

**Background:**

To study the utility of interferon gamma (IFN-γ) as a differentiating biomarker by assessing the aqueous humour and serum of patients with infectious and noninfectious uveitis.

**Methods:**

A total of 40 patients with acute uveitis were divided into 2 groups (18 patients with infectious uveitis and 22 patients with noninfectious uveitis). All the subjects underwent a full ophthalmological examination. Aqueous humour (AqH) and serum samples were collected from uveitis patients. The quantitative levels of IFN-γ in aqueous medium and serum were measured by means of an enzyme-linked immunosorbent assay (ELISA).

**Results:**

The quantitative level of IFN-γ in the aqueous humour was significantly greater (87.5 ± 81) (pg/ml) in infectious uveitis patients than in noninfectious uveitis patients (37.3 ± 9.9) (pg/ml) (*p* value = 0.006). However, the serum IFN-γ level (pg/ml) did not significantly differ between these groups (*p* value = 0.279). Thus, the IFN-γ level in the aqueous humour can be used to discriminate between infectious and noninfectious uveitis with 33.3% sensitivity and 100% specificity.

**Conclusion:**

Aqueous IFN-γ can be used as a biomarker for differentiating between infectious and noninfectious uveitis.

## Background

Uveitis is defined as inflammation of the uveal tissue, which consists of the iris, ciliary body, and choroid. Uveitis frequently involves the impairment of adjacent ocular structures. It has a low incidence, estimated at 17–52/100,000 people per year. However, it accounts for approximately 10% of all cases of blindness worldwide [1]. Uveitis can be caused by infectious agents (infectious uveitis) or it can arise in the absence of any detectable infectious agents (noninfectious uveitis), which can be idiopathic or result from immune-mediated processes or systemic disorders [2].

Identifying the correct treatment approach for uveitis is often a challenge for the ophthalmologist. One of these challenges is that uveitis encompasses a wide variety of pathologies, either systemic or nonsystemic, which may present with a wide range of clinical manifestations, sometimes resulting in a misleading diagnosis and consequently delaying correct treatments [3].

Although the exact pathophysiological mechanism underlying uveitis is not completely understood, the cytokine and chemokine balance appears to play a critical role in the pathway of this entity [4]. Cytokines and chemokines are proteins derived from mononuclear phagocytic cells and T lymphocytes during their growth, differentiation and activation. They regulate all immune and inflammatory responses and are crucial mediators of cytotoxic, humoral, cell-mediated and allergic immunity, and their imbalance could lead to a disturbance in the response of the immune system [5].

Several studies have reported increased levels of cytokines/chemokines in the plasma, vitreous or AqH of uveitis patients. According to many of these studies, the most abundant cytokine detected in both infectious and noninfectious uveitis is IFN-γ [6–14]. Similarly, some cytokines have been shown to induce inflammation in experimental animals after intraocular injection [4].

IFN-γ is a dimeric soluble cytokine. It is a crucial cytokine of the CD4 T helper 1 (Th1) subset and is responsible for the delayed hypersensitivity immune response. In vitro T-cell responses can be evaluated by estimating either the number of IFN-γ-producing T cells or by measuring IFN-γ production via ELISA [15].

We examined AqH and peripheral blood samples from patients with uveitis for IFN-γ. We hypothesized that quantitative level analysis of this cytokine can be used to differentiate between infectious and noninfectious causes of uveitis, especially when the clinical course is challenging.

## Patients and methods

This single-centre prospective, cross-sectional, nonrandomized study included a total of 40 patients with uveitis divided into 2 groups (Group I, Infectious Type, and Group II, Noninfectious Type) who were older than 18 years. Only patients with acute uveitis (according to the SUN criteria) were enrolled. All the patients had already been diagnosed before the samples were taken to avoid overlap in the diagnoses of the patients. We considered aetiological (infectious versus noninfectious) classification rather than anatomical classification because this study aimed to assess the diagnostic value of interferon in aqueous humour, which is not biased by using anatomical classification.

Patients with uveitis receiving systemic medications before samples were taken were excluded. Patients with any ocular or systemic malignancy were also excluded. None of the patients had a recent history of vaccination, including COVID-19 vaccination. All patients were immunocompetent, and none of them were immunocompromised. All patients were enrolled from the Outpatient Ophthalmology Clinic of our affiliated University Hospital from October 2023 to May 2024. All the participants provided informed written consent before enrolment in the study. The clinical study was approved by Al-Azhar’s University Ethics Committee.

All study subjects underwent a complete ophthalmological examination, including best corrected visual acuity (BCVA) measurement using a Snellen chart, slit-lamp biomicroscopy for anterior segment examination, IOP measurement using Goldmann applanation tonometry, and fundus examination.

The diagnosis of herpetic anterior uveitis (AU) and acute retinal necrosis (ARN) in immunocompetent patients was made via the detection of DNA from herpes simplex virus (HSV) or varicella-zoster virus (HZV) in the cellular component of AqH by PCR. Ocular toxoplasmosis was confirmed by increasing serum anti-toxoplasma titres of IgM or IgG in successive serum samples, plus clinical criteria (focal retinitis with severe vitritis) [16]. Cytomegalovirus (CMV) AU in immunocompetent patients was determined by detecting IgM antibodies in the blood sample plus clinical criteria (unilateral coin-shaped dusty KPs, cells and high IOP). Infectious neuroretinitis was determined by an increasing Bartonella IgG titre > 1:64 with a classic history of cat scratch exposure.

Ocular sarcoidosis was diagnosed according to the criteria established at the International Workshop of Ocular Sarcoidosis (IWOS). Behçet’s disease (BD) was diagnosed according to the preferred criteria for BD published by the International Study Group (ISG). HLA-B27-associated AAU was diagnosed by HLA typing of peripheral blood cells plus clinical criteria.

AqH was sampled at the outpatient clinic under a slit lamp after the surface of the cornea and conjunctiva was sterilized with 5% povidone iodine for 3 min and then washed. Approximately 100 μL of AqH was taken from each patient via limbal paracentesis with the use of a 30-gauge needle. AqH samples were aliquoted and frozen at -20 °C for later analysis of gamma-interferon. Serum samples were taken from uveitis patients. One ml of peripheral venous blood was withdrawn and collected in a serum separator tube, allowed to clot for 30 min at room temperature, and centrifuged for 15 min at 1000 × g. Serum was aliquoted and frozen at -20 °C for later analysis of IFN-γ.

Serum and aqueous IFN-γ levels were measured by a quantitative double-antibody sandwich ELISA kit supplied by the Bio-Techne R&D system (USA, Cat. No. DIF50C; AS 1851 Das; Italy (reader) and 16041412 Bio Tek; USA (washer)). All patients underwent standard baseline investigations for erythrocyte sedimentation rate (ESR) and C-reactive protein (CRP) levels.

Data were collected, revised, coded and introduced to a PC using the Statistical Package for Social Science (IBM SPSS) version 23. The data are reported as the means ± standard deviations (SDs). Statistical comparisons of both groups were conducted using *t* tests, with a *p* value < 0.05 considered statistically significant.

## Results

Among the 40 patients initially recruited, 18 patients (8 males and 10 females) were included in the infectious group, with a mean age of 32.2 ± 11.1 years (range: 19–53 years). The noninfectious group included 22 patients (6 males and 16 females), with a mean age of 39.5 ± 12.5 years (range: 22–64 years). The groups did not differ in age or sex (*p* > 0.05) (Table 1).

**Table 1:** Epidemiology and clinical data of both studied groups

Diagnosis	Clinical Data						IFN- γ (pg/ml)	
**I- Infectious Uveitis Group**	**Anatomical type**	**AC Cells**	**Age** (years)	**Gender**	**BCVA**	**IOP** (mmHg)	**Serum** **INF- γ**	**Aqueous INF- γ**
Herpetic AU and ARN	AU and PU	2	38	M	0.01	26	< 15.6	129.2
Herpetic AU and ARN	AU and PU	3	27	M	0.01	24	19.7	219.9
Herpetic AU and ARN	AU and PU	3	28	M	0.01	24	< 15.6	217.9
Herpetic AU	AU	4	48	F	0.02	22	20.7	240.6
Herpetic AU	AU	4	53	F	0.04	18	< 15.6	215.6
Herpetic AU	AU	3	34	F	0.5	20	18.9	130.5
Herpetic AU	AU	4	20	M	0.01	16	24.7	123.3
Ocular Toxoplasmosis	PU	0	17	M	0.1	8	24.5	33.5
Ocular Toxoplasmosis	PU	0	15	M	0.02	10	25.9	35
Ocular Toxoplasmosis	Panuveitis	2	20	M	0.01	4	24.7	23.3
Ocular Toxoplasmosis	Panuveitis	2	34	F	0.2	18	25.8	31
Ocular Toxoplasmosis	Panuveitis	2	29	M	0.2	15	25.3	35
Ocular Toxoplasmosis	Panuveitis	2	34	F	0.2	18	25.8	31
Ocular Toxoplasmosis	PU	1	25	F	0.1	12	20.8	38.5
Ocular Toxoplasmosis	Panuveitis	2	52	F	0.02	12	18.1	38.8
Ocular Toxoplasmosis	Panuveitis	2	35	F	0.01	12	18.4	48.9
CMV anterior uveitis	AU	3	36	F	0.5	22	18.7	94.5
Infectious Neuroretinitis	PU	0	35	F	0.01	12	18.4	48.9
**II-Noninfectious Uveitis Group**								
SLE AU	AU	3	31	F	1	22	33.3	< 15.6
SLE AU	AU	3	32	F	0.5	16	31.5	42.3
SLE AU	AU	4	28	F	0.7	18	33.5	35.3
Behçet’s disease	PU	0	29	M	0.01	12	37.7	26.9
Behçet’s disease	Panuveitis	2	32	M	0.01	14	35.6	38.9
Behçet’s disease	PU	0	27	M	0.01	12	32.9	36.3
Ocular sarcoidosis	AU	3	55	F	0.1	17	< 15.6	37.3
Ocular sarcoidosis	AU and PU	2	55	F	0.1	17	21.1	43.3
Ocular sarcoidosis	AU	4	43	F	0.2	14	18.7	46.7
Ocular sarcoidosis	AU and PU	2	49	F	0.1	15	18.7	45.8
Ocular sarcoidosis	PU	0	51	F	0.1	12	19.5	36.7
Ocular sarcoidosis	AU	2	40	F	0.5	16	18.2	51.1
HLA-B27- associated AAU	AU	2	28	M	0.7	14	20.2	40.8
HLA-B27- associated AAU	AU	4	52	F	0.2	14	21.1	33.2
HLA-B27- associated AAU	AU	2	34	F	0.5	14	20.5	50.1
HLA-B27- associated AAU	AU	2	29	M	0.5	14	18.8	43.7
HLA-B27- associated AAU	AU	2	25	F	0.7	14	20.2	50.8
HLA-B27- associated AAU	AU	2	22	F	0.5	14	23.5	45.3
HLA-B27- associated AAU	AU	3	60	F	0.1	14	16.6	24.4
HLA-B27- associated A AU	AU	3	64	M	0.1	16	16.6	26.5
Fuch’s heterochromic AU	AU	1	40	F	0.5	17	16.5	24.4
Fuch’s heterochromic AU	AU	1	44	F	0.5	18	16.5	25.7

AU: anterior uveitis, PU: posterior uveitis, AAU: acute anterior uveitis, M: male. F: female, INF- γ: Interferon gamma, BCVA: Best Corrected Visual Acuity, AC: Anterior chamber, IOP: Intraocular pressure

For the infectious group, aqueous humour and serum samples were obtained from patients with herpetic AU and ARN (*n* = 3), herpetic AU alone (*n* = 4), cytomegalovirus (CMV) AU (*n* = 1), ocular toxoplasmosis (*n* = 9), and infectious neuroretinitis (*n* = 1). HSV was detected by PCR in aqueous humour samples of the 3 patients with AU with ARN and 2 patients with herpetic AU alone, whereas VZV was detected by PCR in the aqueous humour samples of the other 2 patients with herpetic AU alone.

The noninfectious group included patients with ocular sarcoidosis (*n* = 6), Behçet’s disease (BD, *n* = 3), HLA-B27-associated acute anterior uveitis (AAU, *n* = 8), systemic lupus erythrocytosis (SLE, *n* = 3) and Fuchs heterochromic iridocyclitis (*n* = 2) (Table 1).

A significant decrease in BCVA in the affected eye was detected in the infectious group (mean = 0.11 ± 0.16) compared with that in the noninfectious group (mean = 0.35 ± 0.29) (*p* value = 0.003). However, IOP (mmHg) did not significantly differ between the studied groups (*p* value = 0.8).

The level of aqueous INF-γ (pg/ml) was significantly greater in the infectious group (87.5 ± 81) than in the noninfectious group (37.3 ± 9.9) (*p* value = 0.006). However, the serum INF-γ concentration (pg/ml) did not significantly differ between groups (*p* value = 0.279) (Table 2).

**Table 2:** Comparison of INF-γ (serum and aqueous) concentrations between the studied groups

		Infectious Group (*N* = 18)	Noninfectious Group (*N* = 22)	T	*P* value
Serum INF- γ (pg/ml)	Mean ± SD	21 ± 3.8	23 ± 7.2	10.9	0.279 NS
	Range	15.6–25.9	15.6–37.7		
Aqueous INF- γ (pg/ml)	Mean ± SD	87.5 ± 81	37.3 ± 9.9	**2.88**	**0.006 S**
	Range	23.3-240.6	15.6–51.1		

(T: independent sample T test, S: p value < 0.05 is considered significant, NS: p value > 0.05 is considered nonsignificant)

**Fig. 1:**
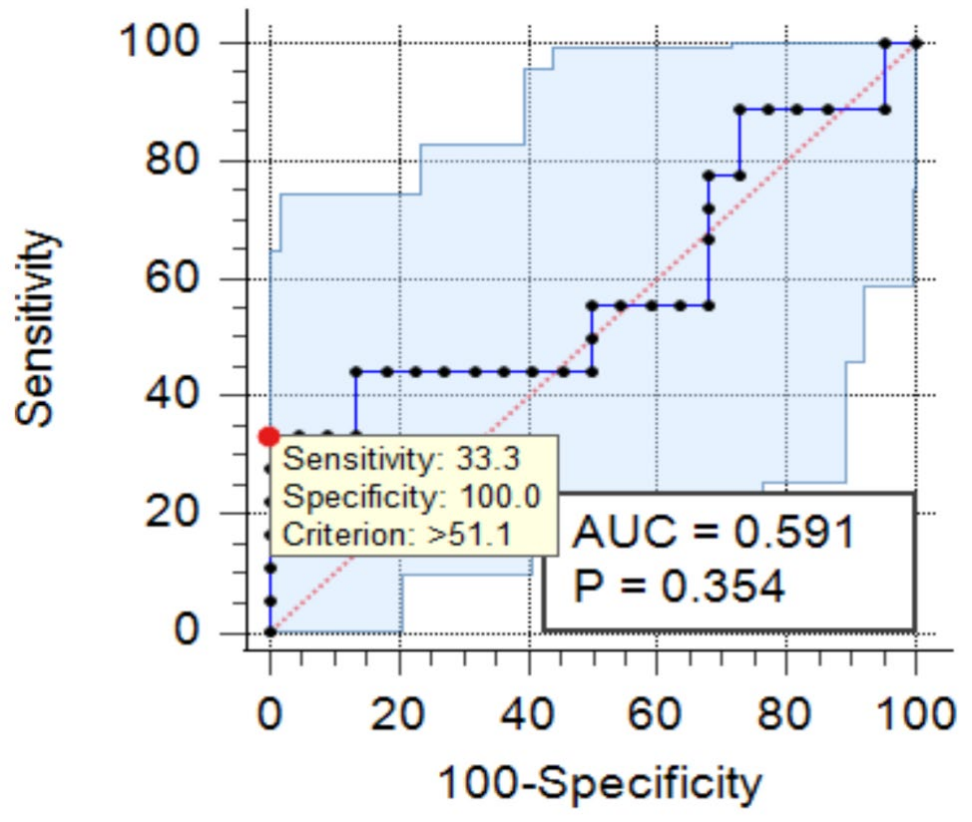
ROC curve between the infectious group and noninfectious group with respect to aqueous INF-γ

An ROC curve revealed that aqueous INF-γ can be used to discriminate between the infectious group and noninfectious group at a cut-off level of > 51.1, with 33.3% sensitivity, 100% specificity, 100% positive predictive value (PPV) and 64.7% negative predictive value (NPV) (*p* value = 0.354) (Table 3) (Fig. 1).

**Table 3:** Diagnostic performance of aqueous INF-γ in discriminating between the studied groups

	Cut off	AUC	Sensitivity	Specificity	PPV	NPV	*p* value
Aqueous INF- γ	> 51.1	0.59	33.3%	100%	100%	64.7%	0.354

(PPV: positive predictive value, AUC: area under the curve, NPV: negative predictive value)

Compared with the other types of infectious uveitis and noninfectious uveitis, the viral uveitis group presented markedly greater aqueous INF-γ production (Table 4).

**Table 4:** Description of aqueous humour INF-γ in patients with uveitis in both studied groups

		Patients with uveitis in both infectious and noninfectious groups (*n* = 40)	
Aqueous INF- γ	> 100	Infectious group	7
		Noninfectious group	0
	< 100	Infectious group	11
		Noninfectious group	22

**Fig. 2:**
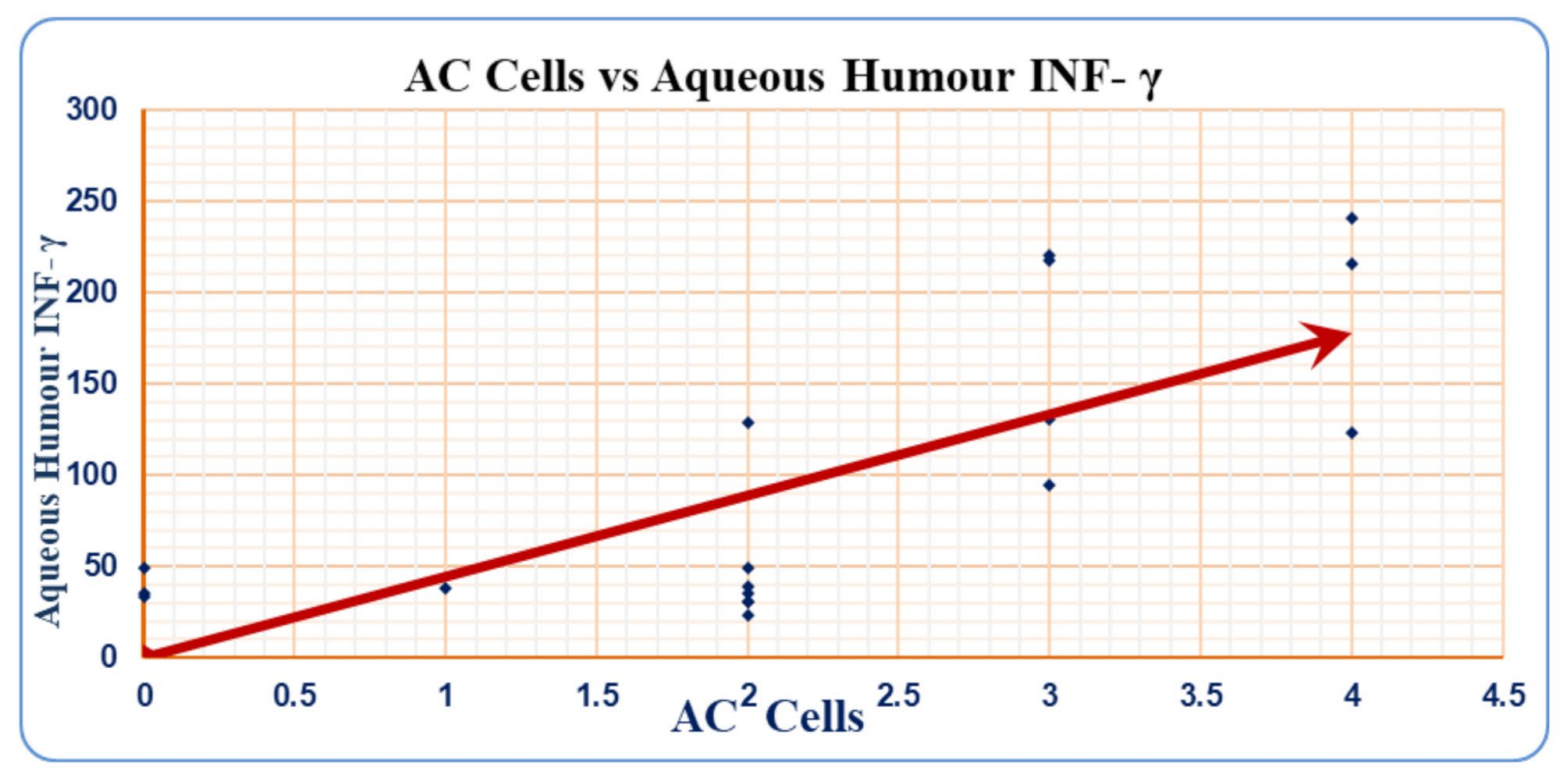
Correlation between AC cells and aqueous humour INF-γ in patients with infectious uveitis

AC cells and aqueous humour INF-γ significantly (*P* = 0.001) and positively (*r* = 0.729) correlated in patients with infectious uveitis (Table 5) & (Fig. 2). However, AC cells and aqueous humour INF-γ did not correlate (*P* = 0.832) in patients with noninfectious uveitis (Table 6). Moreover, serum INF-γ did not correlate with AC cells either patients in the infectious group or those in the noninfectious group (Tables 5 and 6).

**Table 5:** Correlation between AC cells and INF-γ in patients with infectious uveitis

	AC Cells	
	*r*	*P* value
Serum INF- γ	-0.318	0.198
Aqueous INF- γ	0.729	0.001

(r): Pearson correlation coefficient. A P value < 0.05 was considered significant

**Table 6:** Correlation between AC cells and INF-γ in patients with noninfectious uveitis

	AC Cells	
	*r*	*P* value
Serum INF- γ	0.191	0.395
Aqueous INF- γ	-0.048	0.832

(r): Pearson correlation coefficient. A P value > 0.05 was considered nonsignificant

**Fig. 3:**
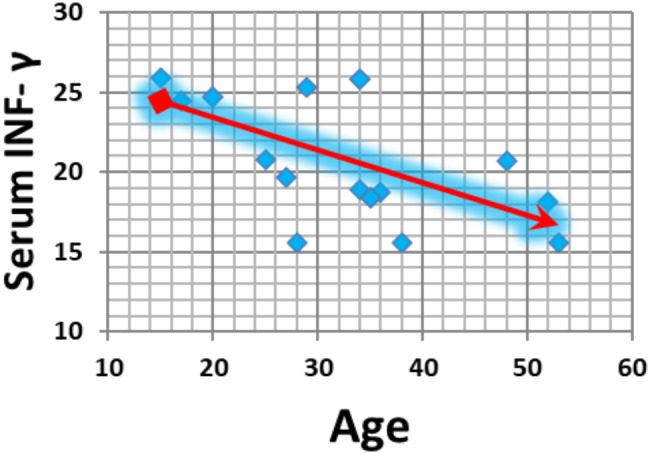
Correlations between the serum INF-γ concentration and age in the infectious group

**Fig. 4:**
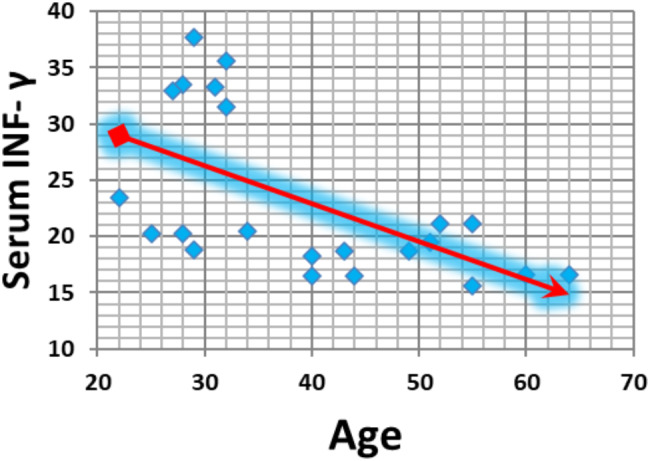
Correlations between the serum INF-γ concentration and age in the noninfectious group

**Fig. 5:**
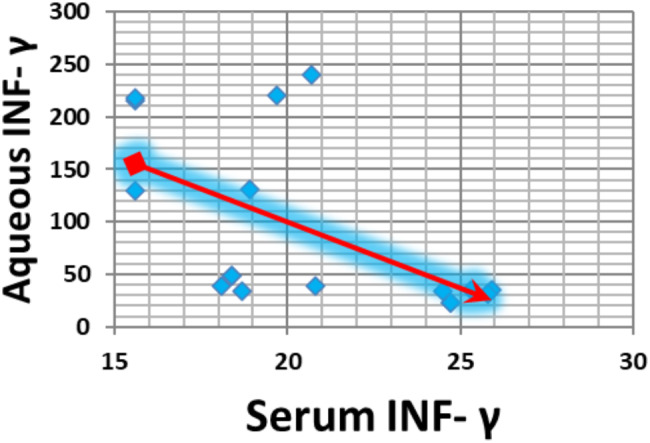
Correlations between the serum γ-INF concentration and aqueous INF-γ concentration in the infection group

The serum INF-γ concentration significantly and negatively correlated with age in both the infectious and noninfectious groups (*p* value = 0.01 and *p* value = 0.004, respectively) (Figs. 3 and 4). Additionally, the serum INF-γ concentration and aqueous humour INF-γ concentration significantly and negatively correlated in the infectious group (*p* value = 0.009) (Fig. 5).

## Discussion

Differentiating between infectious and noninfectious uveitis is highly important because the disease course, outcome, and therapeutic approach are entirely different in each situation. Ocular infections can be significantly worsened in the presence of local or systemic steroids or immunosuppression agents, as these favour increased replication of the infective agent. In addition, these patients may present with atypical manifestations of the disease. Some infectious organisms can also be latent in the eye and reactivated after the introduction of local steroids. For these reasons, infectious uveitis needs to be carefully excluded prior to the administration of steroid or immunosuppressive therapies [17].

Recognition and appropriate work-up of an infectious cause of uveitis and early initiation of targeted anti-infective treatment are important to preserve vision. PCR analysis serves as an important diagnostic tool to establish or exclude the infectious aetiology of uveitis. However, the financial and time-consuming burden of PCR profile analysis for uveitis patients, especially in developing countries, delays the diagnosis and, hence, the early initiation of proper treatment [18].

Thus, an easy, rapid access and screening test for differentiating infectious uveitis from noninfectious uveitis is urgently needed. The focus of many recent studies has been the development of new biomarkers to aid in accurate diagnosis and significantly shift the current management of ocular inflammation [6].

Building on previous studies showing that (IFN-γ) is the most abundant cytokine detected in uveitis [14], our study involved the estimation of the quantitative IFN-γ level in AqH and the sera of patients with infectious and noninfectious uveitis. This level was measured using ELISA. Samples were collected only from patients who had a strictly diagnosed type of uveitis with acute inflammation in the anterior chamber. In addition, none of the patients had been treated with systemic or topical steroids before the samples were taken. Therefore, all patients in each clinical group can be regarded as being in similar condition.

Our results revealed a statistically significant decrease in BCVA in the affected eye in the infectious group (mean = 0.11 ± 0.16) compared with that in the noninfectious group (mean = 0.35 ± 0.29) (*p* value = 0.003). We believe that this finding arose because most cases of infectious uveitis in this study were associated with posterior segment involvement or anterior involvement complicated with high IOP that blurred vision. Therefore, the BCVA during acute attack in infectious uveitis was significantly lower than that in the noninfectious type because of the specific infectious uveitis criteria in those patients rather than the nature of the infectious uveitis in general.

In our study, the INF-γ level in the aqueous humour was significantly greater in the infectious group, with a mean value of 96.4 ± 78.4 (pg/ml) (almost twice as high) than in the noninfectious group (38.3 ± 8.9) (pg/ml) (*p* value = 0.006). However, the serum INF-γ level did not significantly differ between these groups. The INF-γ level in the aqueous humour of patients with viral uveitis was markedly increased, with a maximum value (240.6 pg/ml), compared with that in patients with other types of infectious uveitis and noninfectious uveitis.

These results agree with those of Takase et al. [14], who aimed to determine the cytokine expression profile at the protein level in the AqH and serum of patients with infectious and noninfectious uveitis. The level of IFN-γ in the aqueous humour notably differed between the two groups. The mean IFN-γ level in the aqueous humour of patients with infectious uveitis was 324.5 pg/mL, which was almost 3 times greater than that in patients with noninfectious uveitis (mean, 115.6 pg/mL; *P* = 0.00289). Additionally, the level of INF-γ in the aqueous humour of ARN patients was markedly increased, with a maximum of 571.4 pg/ml, compared with that in patients with other types of infectious uveitis and noninfectious uveitis. However, in this study, the infectious uveitis cases involved were all viral uveitis cases. This fact explains the greater mean IFN-γ level in the aqueous humour of patients in the infectious uveitis group in this study. IFN-γ was detected in the sera of half of the patients with infectious uveitis, although the concentrations were close to the detection limit.

On the other hand, Lacomba et al. [4] assessed the cytokine profile in the AqH and peripheral blood from patients with uveitis and controls. The IFN-γ and IL-2 levels in the aqueous humour and serum were greater in samples from patients with uveitis than in those from controls, but the IFN-γ levels in the peripheral blood (39.26 ± 34.11 pg/ml) were greater than those in the aqueous humour (13.86 ± 1.68 pg/ml) among these patients. This difference may be due to the variance in the cause, severity, duration and pattern of uveitis in their study compared with the control. In addition, most uveitis cases in that study were due to noninfectious causes.

Based on cytokine/chemokine profile analysis, many studies have reported increased levels of IFN-γ in either the plasma or AqH of uveitis patients compared with controls [6–14]. To our knowledge, this study is the first to evaluate aqueous and serum levels of IFN-γ alone in patients with infectious and noninfectious uveitis.

Because the IFN-γ level in the aqueous humour is specific rather than sensitive, aqueous IFN-γ measurement might be proposed in parallel with the gold standard PCR for differentiating infectious from noninfectious uveitis. A high aqueous IFN-γ level should be considered as an additional indicator for infectious uveitis, especially in the case of negative PCR results.

## Conclusion

We found a significant increase in aqueous humour IFN-γ levels in patients with infectious uveitis. Aqueous humour INF-γ can be used to discriminate between these two groups with 33.3% sensitivity and 100% specificity. Understanding the role of IFN-γ in ocular inflammation may provide new clues for the management of uveitis. Measurements of IFN-γ levels in the AqH may also play a role in predicting relapses and monitoring disease activity in patients with uveitis.

The main limitation of this study is the small sample size, which is not sufficient to determine the all infectious and noninfectious causes of uveitis. Also, we depended on the aqueous IFN-γ levels in all anatomical types of uveitis and did not consider the vitreous IFN-γ levels in PU and panuveitis cases. Further studies involving IFN-γ analysis in conjunction with clinical and therapeutic data are needed. In addition, more studies of IFN-γ analysis for each disease individually in infectious and noninfectious causes would add more insights into the role of this cytokine in uveitis management.

## Data Availability

No datasets were generated or analysed during the current study.

## Abbreviations

AAU: Acute anterior uveitis
AC: Anterior chamber
AqH: Aqueous humour
ARN: Acute retinal necrosis
AU: Anterior uveitis
BCVA: Best corrected visual acuity
BD: Behçet’s disease
CMV: Cytomegalovirus
CRP: C-reactive protein
ELISA: Enzyme-linked immunosorbent assay
ESR: Erythrocyte sedimentation rate
HSV: Herpes simplex virus
IFN-γ: Interferon Gamma
IOP: Intraocular pressure
ISG: International study group
IWOS: International Workshop of ocular sarcoidosis
PCR: Polymerase chain reaction
Th1: T helper1
VZV: Varicella zoster virus
